# Excess risk of preterm birth with periconceptional iron supplementation in a malaria endemic area: analysis of secondary data on birth outcomes in a double blind randomized controlled safety trial in Burkina Faso

**DOI:** 10.1186/s12936-019-2797-8

**Published:** 2019-05-06

**Authors:** Bernard Brabin, Sabine Gies, Stephen A. Roberts, Salou Diallo, Olga M. Lompo, Adama Kazienga, Loretta Brabin, Sayouba Ouedraogo, Halidou Tinto

**Affiliations:** 1 Clinical Division, Liverpool School of Tropical Medicine, and Institute of Infection and Global Health, University of Liverpool, Liverpool, UK; 2 Global Child Health Group, Academic Medical Centre, University of Amsterdam, Amsterdam, The Netherlands; 3 Department of Biomedical Sciences, Prince Leopold Institute of Tropical Medicine, Antwerp, Belgium; 4 Medical Mission Institute, Würzburg, Germany; 5 Centre for Biostatistics, Division of Population Health, Health Services Research and Primary Care, Faculty of Biology, Medicine and Health, University of Manchester, Manchester Academic Health Science Centre (MAHSC), Manchester, UK; 6 Institute for Research in Health Sciences-Clinical Research Unit of Nanoro, (IRSS-URCN), Nanoro, Burkina Faso; 7 Service d’Anatomocytopathologie et de Médicine Légale, Centre Hospitalier Universitaire Yalgado Ouedraogo, Ouagadougou, Burkina Faso; 8 Division of Cancer Sciences, School of Medical Sciences, Faculty of Biology, Medicine and Health, University of Manchester, Manchester Academic Health Science Centre (MAHSC), Manchester, UK

**Keywords:** Iron supplements, Preterm birth, Fetal growth, Malaria, Adolescents, Burkina Faso

## Abstract

**Background:**

Iron supplementation before a first pregnancy may improve the future health of mother and baby by reducing maternal anaemia. Iron supplementation could, however, increase malaria infections, notably in primigravidae who are most susceptible. The pathogenicity of other iron-utilizing pathogens could also increase, causing inflammation leading to increased risk of adverse birth outcomes. This paper reports pre-specified secondary birth outcomes from a safety trial in Burkina Faso in an area of high malaria endemicity. Primary outcomes from that trial had investigated effects of long-term weekly iron supplementation on malaria and genital tract infections in non-pregnant and pregnant women.

**Methods:**

A double-blind, randomized controlled trial. Nulliparous, mainly adolescent women, were individually randomized periconceptionally to receive weekly either 60 mg elemental iron and 2.8 mg folic acid, or 2.8 mg folic acid alone, continuing up to the first antenatal visit for those becoming pregnant. Secondary outcomes were ultrasound-dated gestational age, fetal growth, placental malaria, chorioamnionitis and iron biomarkers. Seasonal effects were assessed. Analysis was by intention to treat.

**Results:**

478 pregnancies occurred to 1959 women: 258/980 women assigned iron and folic acid and 220/979 women assigned folic acid alone. Malaria prevalence at the first antenatal visit was 53% (iron) and 55% (controls). Mean birthweight was 111 g lower in the iron group (95% CI 9:213 g, P = 0.033). Mean gestational ages were 264 days (iron) and 269 days (controls) (P = 0.012), with 27.5% under 37 weeks compared to 13.9% in controls (adjRR = 2.22; 95% CI 1.39–3.61) P < 0.001). One-third of babies were growth restricted, but incidence did not differ by trial arm. Half of placentae had evidence of past malaria infection. C–reactive protein > 5 mg/l was more common prior to births < 37 weeks (adjRR = 2.06, 95% CI 1.04–4.10, P = 0.034). Preterm birth incidence during the rainy season was ~ 50% in the iron arm and < 20% in controls (P = 0.001). Chorioamnionitis prevalence peaked in the dry season (P = 0.046), with no difference by trial arm (P = 0.14).

**Conclusion:**

Long-term weekly iron supplementation given to nulliparous women in a malaria endemic area was associated with higher risk of preterm birth in their first pregnancy.

*Trial Registration* NCT01210040. Registered with Clinicaltrials.gov on 27th September 2010

**Electronic supplementary material:**

The online version of this article (10.1186/s12936-019-2797-8) contains supplementary material, which is available to authorized users.

## Background

Giving iron to young women ahead of their first pregnancy could potentially improve the future health of both mother and baby by reducing maternal anaemia [1–3], providing iron-infection interactions are negligible. Iron supplementation may increase malaria susceptibility [4], especially in primigravidae who are at highest risk [5]. It may also increase pathogenicity of other iron-utilizing organisms, such as with reproductive tract infections, causing inflammation and increasing the risk of preterm delivery and low birthweight. Gram-negative bacteria, causative agents of chorioamnionitis, utilize multiple iron-uptake mechanisms as does bacterial vaginosis (BV), associated with polymicrobial vaginal biofilms [6].

Existing systematic reviews on iron supplementation in pregnancy include few studies from malaria endemic areas, and those cited are from locations with no, or very little malaria [7]. A safety trial was conducted in young women in Burkina Faso in an area of high malaria endemicity to investigate the effects of periconceptional iron supplementation on malaria and genital tract infection risk in non-pregnant and pregnant women [8, 9]. This safety trial measured malaria prevalence at first antenatal visit (ANC1) as the primary (non-inferiority) outcome, and reported that weekly supplementation did not increase malaria risk (risk ratio 1.00, 95% CI 0.97–1.03), or improve iron status (iron deficiency risk ratio 0.84 (0.46–1.54), or reduce anaemia (risk ratio 0.96, 0.83–1.10) in these young, mostly adolescent menstruating women [8]. Genital tract infection markers were also assessed [9]. BV, *Trichomonas vaginalis* prevalence and microbiota profiles did not differ at trial end-points although at baseline, iron replete participants were less likely to have normal vaginal flora. At their first antenatal study visit weekly supplementation was withdrawn and replaced by haematinics for all women regardless of allocation. Women were followed to delivery and the trial protocol included pre-specified (superiority) secondary birth outcomes, which are reported in this paper along with exploratory analyses of the impact of infection, inflammation, seasonality and placental pathology on these outcomes.

## Methods

The trial protocol and amendments were approved by ethical review boards and regulatory authorities at each collaborating centre. This birth outcome analysis was conducted within a randomized trial of the safety of weekly iron and folic acid supplementation in young women exposed to malaria [8, 9]. Specified birth outcomes were: gestational age, preterm birth (PTB), small for gestational age and low birthweight. Additional summary data on the main trial, the primary malaria outcome measures, measurements of iron biomarkers, and other details relevant to this paper, are provided in Additional file 1.

### General procedures

Between April 2011 and January 2014, a randomized, double blind, controlled trial was conducted amongst nulliparous, non-pregnant residents aged 15–24 years in a hyper-endemic malaria endemic area of Burkina Faso. HIV prevalence is low and reported as 1.2% among women aged 15–49 years and 0.76% among pregnant women [10]. Syphilis sero-prevalence among first time female blood donors is also low in Burkina Faso (1.1%) [11]. The study was undertaken within the rural Nanoro Health and Demographic Surveillance system which has a population of approximately 55,000, and area of 600 km^2^ [12]. The dry season is from October to May and wet season from June to September, with malaria peak prevalence between July to September [13].

Two cohorts of supplemented women were followed—women remaining non-pregnant (not reported here) and women who experienced pregnancy during, or shortly after the 18 month supplementation period. Participants were individually randomized to receive either a weekly capsule containing ferrous gluconate (60 mg) and folic acid (2.8 mg), or an identical capsule containing folic acid alone (2.8 mg) (see Additional file 1 for randomization details). The regimen followed World Health Organization (WHO) guidelines, updated in 2016 [1, 2]. The primary outcome result of *Plasmodium falciparum* parasitaemia prevalence at ANC1, and secondary outcome results on lower genital infections have been reported [8, 9]. Weekly iron supplementation did not increase malaria risk at the first antenatal visit [8].

At enrolment, and at two further antenatal visits, a venous blood sample was collected for plasma ferritin, serum transferrin receptor (sTfR) and C-reactive protein (CRP) measurements. Pregnant women were referred for assessment at 13–16 weeks gestation (ANC1) as indicated by the last menstrual period which was obtained from weekly review during follow-up of the non-pregnant cohort. Gestational age was estimated by ultrasound examination at ANC1 with a FF Sonic UF-4100 (Fukuda Denshi) scanner. Gestational age was estimated by crown rump length in the first trimester and by biparietal diameter, femur length and abdominal circumference afterward. A blood sample was obtained for measurement of haemoglobin, iron biomarkers, and malaria screening as malaria end-points were the primary outcome measure for the trial [8]. A syphilis test was done (RPR VDRL Carbon, ELITechGroup). A self-taken vaginal swab was requested for *Trichomonas vaginalis* screening by qPCR, and slides were screened for BV as previously reported [9]. Symptomatic women were treated for BV and *Trichomonas vaginalis.*

Weekly supplements were withdrawn at ANC1 and replaced by haematinics for all pregnant women regardless of allocation according to national policy (60 mg iron, 400 µg folic acid daily). A second antenatal study visit (ANC2) was scheduled between 33 and 36 weeks. A single measure of blood pressure was taken at ANC1 and ANC2 with explanation to the mother who rested 5 min before use of the sphygmomanometer (Riester). Weekly follow-up continued till delivery, and adherence was recorded [8]. All women received a first dose of intermittent preventive treatment with sulfadoxine-pyrimethamine (IPTp-SP) at ANC1 if gestational age was > 13 weeks. Women ≤ 13 weeks gestation, if positive for malaria by rapid diagnostic test (RDT) (Bioline SD, Malaria Antigen Pf), were treated with oral quinine. A second scheduled IPTp-SP dose was provided through routine antenatal care. At ANC2 women were encouraged to deliver at Nanoro Hospital or the nearest Health Centre, where free obstetric care was provided by the study. The main analyses are confined to those who delivered within the study area and excluded births from migrated women who delivered elsewhere.

Adverse events (SAEs), including maternal deaths, were collected by active (weekly) and passive surveillance and reported according to available information from field workers, health centre, and hospital staff. These events are reported separately and previously published [8]. A summary is provided in Additional file 1.

### Assessment methods

Study nurses examined babies within 24–48 h of delivery, and recorded birthweight on an electronic scale to within 10 g (SECA 384, Hamburg, Germany, precision ± 5 g for weights < 5000 g, ± 10 g above 5000 g), and clinically assessed gestational age [14]. Following hospital delivery placental biopsies (2.5 × 1 cm) were excised from fetal and maternal sides at mid-distance between umbilical cord insertion and the placental border, and placed in 10% neutral buffered formalin. Laboratory procedures for iron biomarker assays have been previously reported [9, 15], and details on placental histopathology procedures are available in Additional file 1. Classification of placental malaria was based on the presence of hemozoin and parasitized red blood cells in the inter-villous space [16]. Severity of acute chorioamnionitis and funiculitis (acute histologic chorioamnionitis) was graded histologically as early (grade 1), intermediate (grade 2) and advanced (grade 3) following the Redline-classification [17, 18].

### Statistical analysis

The sample size was determined from formal power calculations for the malaria trial endpoints [9]. Primary analyses presented here are comparisons of the four pre-specified secondary birth outcomes of birthweight, gestation, PTB and placental malaria in singleton babies by trial arm on an intention to treat basis. Iron and inflammation biomarkers, and placental chorioamnionitis were pre-specified exploratory outcomes. PTB was defined as a livebirth or stillbirth that took place at least 20 but before 37 completed weeks; early preterm as between 20 and 33 completed weeks; post-term after 41 completed weeks; low birthweight as less than 2500 g; and miscarriage as spontaneous loss of a probable/clinical pregnancy before 20 weeks of gestational age. Fetal growth restriction (SGA) was defined as birthweight below the 10th centile for gestation and gender, indicated by standard reference data [19].

Prevalence analyses of treatment effects (intention to treat [ITT] analysis of treatment allocation) based on data at specific time points utilized risk-ratio binomial models unadjusted and adjusted for mid-upper-am-circumference (MUAC) at baseline, bed net use (proportion of weekly visits where a bed net was reported to have been used the previous night) to ANC1 or last follow-up, and birth month as a categorical variable. CRP was analysed using analogous ordinary regression models following logarithmic transformation and adjusted using the calendar month of assessment. Results are expressed as risk-ratios (or density/biomarker level ratios) with 95% CI and associated likelihood-ratio significance level.

Similar models were used to compare CRP and placental pathology between women whose babies were term/preterm or SGA/AGA (small/appropriate for gestational age), pooling the trial arms and with and without adjusting for assessment (CRP) or birth (placental markers) month. A non-linear sinusoidal cyclic model, based on calendar month of assessment, with a 12 month period and adjusting for baseline MUAC and bed net use, as above, was used to formally test for seasonality and for differential magnitude of seasonal effects between arms.

P-values were determined using Likelihood-ratio tests comparing appropriately reduced models. Sensitivity analyses confirmed the adequacy of this seasonal model. Statistical significance was two-sided at alpha = 0.05. No formal adjustment is made for multiple testing as the outcomes examined were expected to be highly correlated. Other pre-specified birth outcomes related to congenital anomalies and perinatal deaths have been previously reported [8]. All analyses were performed using R statistical environment version 3.3 [20].

## Result

### Participants

**Fig. 1:**
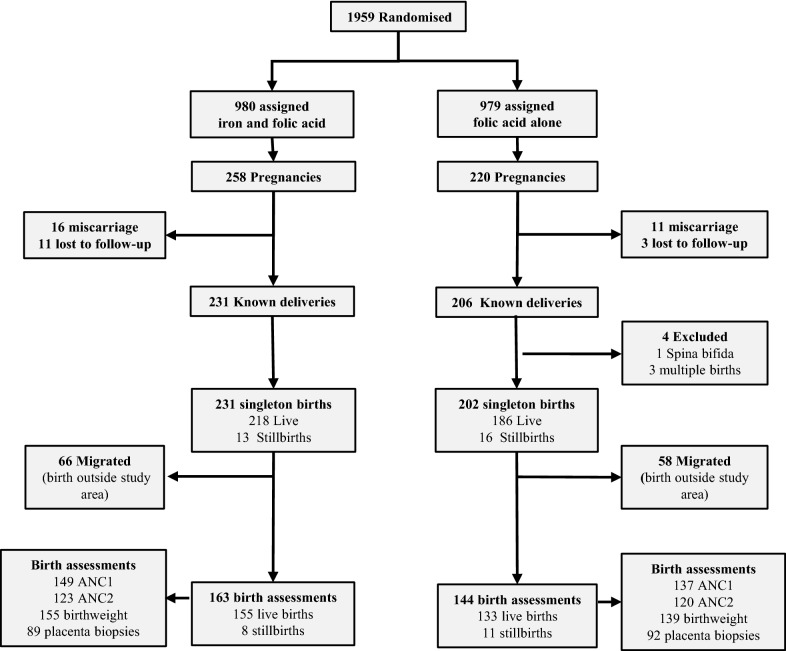
Participant flow diagram. *ANC* antenatal care

Of 1959 nulliparous women, 980 were randomly assigned weekly supplements with iron and folic acid, and 979 folic acid supplements alone. During follow-up 478 pregnancies occurred, with 437 known deliveries (Fig. 1). Following exclusions due to miscarriages, severe congenital abnormalities and multiple births, there were 433 singleton births. After losses due to out-migration, 307 births, (288 live births, 19 stillbirths) were included in the primary analyses presented here. Demographic, nutritional and clinical characteristics were comparable between intervention and control groups, with adolescents (< 20 years) comprising 90.9% of enrolled participants (Table 1). Characteristics of excluded women were similar to those providing birth outcomes (Additional file 2: Table S1). Frequency of pregnancy was higher in the iron group (P = 0.052). Prevalence of undernutrition (BMI < 18.5 kg/m^2^) was 18.6%.

**Table 1:** Baseline characteristics of nulliparae with birth assessments by intervention group

Characteristic	Iron	Control
Sample size	163	144
Demographic and socioeconomic		
Mean age, years [IQR]	17.0 [16.0–18.0]	17.0 [16.0–18.0]
Age < 20, n (%)	145/163 (89)	134/144 (93)
Age < 17, n (%)	62/163 (38)	65/144 (45)
Reproductive Age, years [IQR]	4 [3–5]	3 [2–4]
Ethnic Group Mossi, n (%)	159/163 (98)	141/144 (98)
Religion		
Muslim	49/163 (30)	36/143 (25)
Christian	97/163 (53)	66/143 (56)
Traditional	27/163 (17)	41/143 (29)
No education, n (%)	109/162 (67)	91/144 (63)
Primary education, n (%)	34/162 (21)	22/144 (15)
Lower and higher secondary, n (%)	19/162 (12)	31/144 (22)
Literate, n (%)	45/161 (28)	46/143 (32)
Occupation		
Student^a^	41/163 (25)	39/144 (27)
Trading	7/163 (4)	7/144 (5)
Domestic	101/163 (62)	74/144 (51)
Farmer	75/163 (46)	58/144 (40)
Unmarried, n (%)	145/163 (89)	133/144 (92)
Bed net use to ANC1, %, median [IQR]^b^	63 [50–83] [2 missing]	67 [47–84] [1 missing]
Clinical		
Menarcheal, n (%)	155/163 (95)	133/144 (92)
Sexually active, n (%)	67/163 (41)	48/144 (33)
Height, cms [IQR]	160 [156–164]	159 [155–163]
Weight, kg [IQR]	51.4 [47.8–54.7]	50.9 [46.7–55.5]
BMI, kg/m^2^ [IQR]	20.0 [18.9–21.1]	20.2 [18.9–21.0]
BMI < 18.5 kg/m^2^, n (%)	29/163 (18)	28/144 (19)
MUAC, cms [IQR]	24.1 [22.9–25.2]	24.2 [22.8–25.4]
Iron biomarkers		
Median Plasma CRP, mg/l [IQR]	0.59 [0.25–1.62] [5 missing]	0.68 [0.24–1.70] [2 missing]
CRP > 5 mg/l, n (%)	13/158 (8)	14/142 (10)
CRP > 10 mg/l, n (%)	5/158 (3)	7/142 (5)
Median ferritin, µg/l [IQR]	51.50 [28.00–78.75] [4 missing]	46.00 [24.00–85.00] [3 missing]
Median sTfR, mg/l [IQR]	6.13 [5.06–7.63] [3 missing]	6.33 [5.22–8.31] [3 missing]
Median sTfRr/log ferritin ratio	3.72 [2.89–5.25] [4 missing]	3.84 [2.84–5.83] [3 missing]
Iron deficiency (adj ferritin), n (%)^c^	16/157 (10) [6 missing]	21/141 (15) [3 missing]
Iron deficiency (sTfR/log ferritin), n (%)^d^	33/159 (21) [4 missing]	42/141 (30) [3 missing]
Antenatal care		
First study visit (ANC1)	149/163 (91)	137/144 (95)
Second study visit (ANC2)	123/163 (76)	120/144 (83)
Median total ANC visits [IQR]^e^	4.00 [3.00–5.00]	4.00 [3.00–5.00]
Median IPTp doses, IQR	2.00 [1.00–2.00]	2.00 [2.00–2.00]
≥ One IPTp dose n/N (%)	153/163 (94)	140/144 (97)
≥ Two IPTp dose, n/N (%)	109/163 (67)	114/144 (79)

*IPTp* Intermittent preventive treatment with sulfadoxine-pyrimethamine

^a^Domestic labour and farming not mutually exclusive

^b^Bed net use is the percentage of weekly visits between enrolment and ANC1 where bed nets were reported being used the night before

^c^Ferritin < 15 μg/L if CRP < 10 mg/l, or ferritin < 70 μg/L if CRP ≥ 10 mg/l. Ranges for normal controls were: ferritin, 69.1–114.7 µg/l; sTfR, 4.2-5.9 mg/l; CRP, 5–8 mg/l

^d^Ratio of sTfR (mg/l) to log_10_ ferritin (µg/l) > 5.6

^e^ANC1, ANC2 and non-study ANC visits

Mean gestational age at ANC1 was 18.4 ± (SD) 5.9 weeks and at ANC2, 34.2 ± 1.6 weeks. At ANC1 malaria parasitaemia prevalence was 53% in the iron arm and 55% in controls [8]. Iron deficiency prevalence at ANC1, based on the sTfR/log_10_ ferritin ratio, was 11% (iron, 17/149) and 13% (controls, 18/135) (P = 0.53, adjusted for season, baseline MUAC and bed net use). At ANC2 prevalence was 28% (iron, 34/122) and 28% (controls, 34/118), (P = 0.91). Mean haemoglobin concentration was similar in both trial arms at ANC1 (iron 10.5 g/dl ± 1.4; control 10.5 ± 1.5 g/dl), and at ANC2 (iron 10.7 g/dl ± 1.4; control, 10.8 ± 1.4). There were fewer ANC2 visits in the treated arm (76% versus 83%, P = 0.094), which received a slightly lower number of mean IPTp-SP anti-malarial doses (67% of iron, receiving 2 or more doses compared to 79% of controls, P = 0.021) (Table 1). The median total number of antenatal visits (ANC1, ANC2, and non-study visits) was four, and identical between trial arms. Two women had mild hypertension at ANC1 (both controls) (diastolic blood pressure 90–99 mmHg, systolic 140–149 mmHg), and none were hypertensive at ANC2. There was no difference in mean height change between baseline and ANC1 between groups (iron, 1.3 ± (SD) 1.2 cm; control 1.3 ± 1.5 cm, P = 0.83).

### Birth outcomes by trial arm

**Fig. 2:**
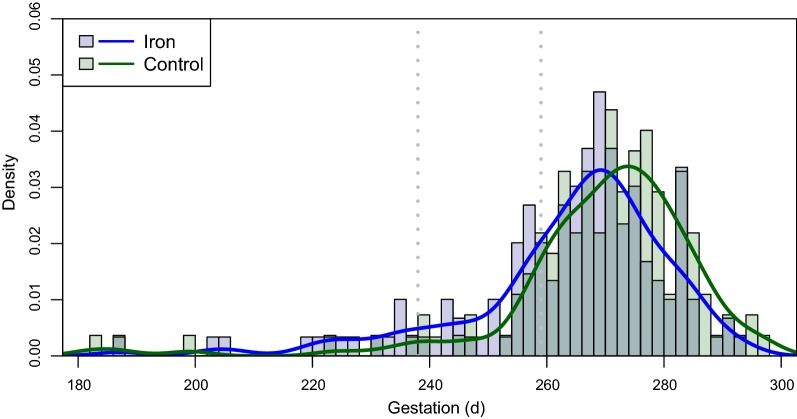
Gestational age distribution in days of livebirths in iron and control arms. Vertical stippled lines indicate 43 weeks and 37 weeks gestation

**Fig. 3:**
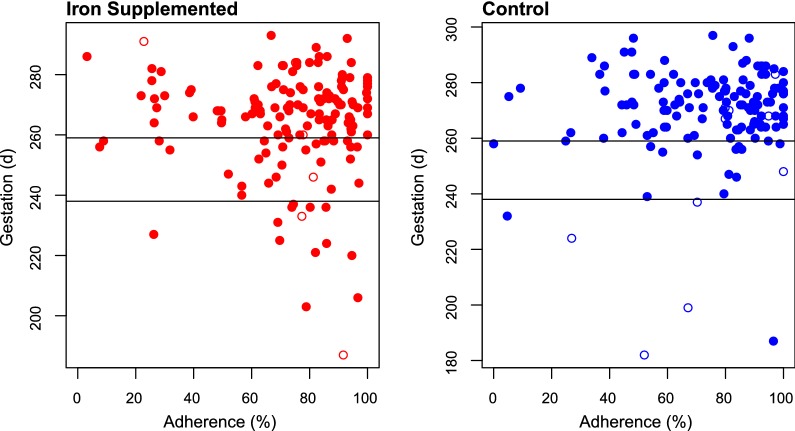
Gestation at birth by directly observed treatment adherence based on percentage of scheduled treatments received. Upper horizontal line: 37 weeks gestation; lower horizontal line 34 weeks gestation. Stillbirths plotted with open symbols

The mean birthweight was 100 g lower in the iron group (95% CI 205:5), P_adj_ = 0.033), with a greater risk of LBW (OR: 1.34 (0.99–1.81) of borderline significance (P = − 0.062) (Table 2). Mean gestational age at birth was 264.0 days in iron-supplemented women and 269.4 days in controls (P_adj_ = 0.012). In the iron arm, the whole distribution curve for gestational age at delivery in livebirths was shifted to the left (Fig. 2). For iron-supplemented women PTB was more frequent at both < 37 weeks (27.5% vs 13.9%; P_adj_ < 0.001) and < 34 weeks (9.4% *vs* 4.4%; P_adj_ = 0.069), (Table 2). The 6 stillbirths of known gestation born to mothers receiving iron had a mean gestation of 250 days and of these, 3 (50%) were < 37 weeks and 4 intrapartum. In the control arm 10 stillbirths had a mean gestation of 245 days and 5 (50%) were < 37 weeks and 7 intrapartum. In 13 of the 14 PTB births < 34 weeks in supplemented women, tablet adherence was between 69% and 100%, compared to only 2 of the 6 in the control group (Fig. 3).

**Table 2:** Birth outcomes by trial arm

Outcome^a^	n	missing	Iron	Control	Relative risk/difference^b^	P	Adjusted relative risk/difference^c^	P_adj_^c^
Infant								
Neonatal death, n/N (%)^d^	433	0	6/231 (2.6)	3/202 (1.5)	1.75 (0.44; 6.93)	0.41	1.70 (0.43;6.80)	0.44
Livebirth male, n/N (%)^d^	409	24	106/219 (48.4)	101/190 (53.2)	0.91 (0.75; 1.10)	0.34	0.89 (0.74;1.08)	0.25
Gestation, days ± SD^e^	286	21	264.0 ± 17.5 [14 missing]	269.4 ± 17.3 [7 missing]	− 5.36 (− 9.42; − 1.30)	0.010	− 5.21 (− 9.26; − 1.16)	0.012
Preterm < 37 weeks, n/N (%)^e^	286	21	41/149 (27.5)	19/137 (13.9)	1.98 (1.21; 3.25)	0.004	2.24 (1.39; 3.61)	< 0.001
Preterm < 34 weeks, n/N (%)^e^	286	21	14/149 (9.4)	6/137 (4.4)	2.15 (0.85; 5.45)	0.091	2.25 (0.89; 5.67)	0.069
Post-term > 41 weeks, n/N (%)^e^	286	21	4/149 (2.7)	9/137 (6.6)	0.41 (0.13; 1.30)	0.11	0.41 (0.13; 1.31)	0.11
Birthweight, g ± SD^e^	294	13	2640 ± 486 [8 missing]	2740 ± 420 [5 missing]	− 100 (− 205; 5)	0.062	− 111 (− 213; − 9)	0.033
Low birthweight, n/N (%)^e^	294	13	54/155 (34.8)	40/139 (28.8)	1.21 (0.86; 1.70)	0.27	1.34 (0.99; 1.81)	0.062
SGA, n/N (%)^e^	277	30	41/143 (28.7)	48/134 (35.8)	0.80 (0.57; 1.13)	0.20	0.82 (0.59; 1.14)	0.25
Placental pathology, n/N (%)^f^								
Acute malaria	181	0	5/89 (5.6)	7/92 (7.6)	0.74 (0.24; 2.26)	0.59	0.84 (0.28; 2.53)	0.76
Chronic malaria	181	0	28/89 (31.5)	20/92 (21.7)	1.45 (0.88; 2.38)	0.14	1.30 (0.84; 1.99)	0.25
Past malaria	181	0	44/89 (49.4)	52/92 (56.5)	0.87 (0.66; 1.16)	0.34	0.89 (0.71; 1.11)	0.29
Chorioamnionitis, grade 3^g^	179	2	6/89 (6.7)	7/90 (7.8)	0.87 (0.30; 2.50)	0.79	0.87 (0.3; 2.51)	0.80
Chorioamnionitis, grade 2 and 3	179	2	36/89 (40.4)	44/90 (48.9)	0.83 (0.59; 1.15)	0.26	0.75 (0.58–0.97)	0.050

^a^Gestational age is by ultrasound at ANC1 except one by Ballard assessment at birth; Preterm: 20–36 completed weeks, or 20 to 33 completed weeks

^b^Relative risk for the categorical variables; difference in days or g for gestation and weight

^c^Adjusted for MUAC at baseline, bed net use to ANC1 or last follow-up, and birth month

^d^Based on full analysis dataset, n = 435

^e^Based on 307 with post-delivery assessment

^f^Based on 181 with a placental biopsy. Acute infection: only parasites and minimal hemozoin deposition in the macrophages but not fibrin

Chronic infection: parasites and hemozoin deposition; Past infection: hemozoin usually mixed with fibrin but no parasites

^g^Severity of acute chorioamnionitis and funiculitis (acute histologic chorioamnionitis) was graded histologically as early (grade 1), intermediate (grade 2) and advanced (grade 3) following the Redline-classification [17, 18]

Almost one-third of babies were growth restricted (SGA), but incidence did not differ significantly by trial arm (Table 2). Nine neonatal deaths occurred—six were babies of women who received iron. Approximately half of all placentae had histological evidence of past malaria infection, and chronic placental malaria was more frequent in women receiving iron although this difference did not reach statistical significance (adjusted RR = 1.30, P_adj_ = 0.25, Table 2). Grade 2 or 3 chorioamnionitis commonly occurred (mean 44.7%), with marginally lower prevalence in women receiving iron (P_adj_ = 0.050).

### Association between birth outcomes, inflammation and infection

Plasma CRP concentrations did not differ between trial arms at either ANC1 or ANC2 (Additional file 2: Table S2). Therefore the two arms were pooled for exploratory analyses of the associations between inflammation, PTB and SGA. Mean CRP concentrations were significantly raised in women with PTB, with high values (> 5 mg/l) more prevalent at both ANC1 (adjusted risk ratio 1.60, P = 0.044), and ANC2 (adjusted risk ratio 2.06, P = 0.034) after adjustment for assessment month (Table 3). Values > 10 mg/l were also more prevalent at ANC1 (P = 0.036) and ANC2 (P = 0.26), (Table 3). Mean maternal CRP concentrations were increased with SGA outcomes, with values > 5 mg/l less frequent at ANC1 amongst mothers of SGA babies (adjusted RR = 0.62, P = 0.009) (Table 3). CRP values at ANC2 or at baseline did not differ significantly between women with SGA compared to AGA outcomes.

**Table 3:** C-reactive protein concentration at baseline, ANC1, or ANC2, for preterm/term or SGA/AGA outcomes

Parameter mg/l	n	Outcome^a^		Difference or Relative risk^a^ (95% CI)	P	Adjusted difference or relative risk^b^ (95% CI)	P_adj_^c^
		Term	Preterm				
Baseline							
Log CRP, ± SD	280	− 0.2 ± 0.7	− 0.09 ± 0.7	1.18 (0.85; 1.63)	0.32	1.20 (0.87; 1.65)	0.26
CRP > 5, n/N (%)	280	20/221 (9)	6/59 (10)	1.11 (0.53; 2.33)	0.79	1.14 (0.55; 2.35)	0.74
CRP > 10, n/N (%)	280	8/221 (4)	4/59 (7)	1.62 (0.70; 3.75)	0.31	1.65 (0.73; 3.74)	0.31
ANC1							
Log CRP, ± SD	282	0.6 ± 0.7	0.7 ± 0.7	1.28 (0.91; 1.80)	0.15	1.33 (0.94;1.89)	0.097
CRP > 5, n/N (%)	282	104/223 (47)	36/59 (61)	1.59 (0.99; 2.54)	0.049	1.60 (1.00; 2.55)	0.044
CRP > 10, n/N (%)	282	69/223 (31)	26/59 (44)	1.55 (0.99; 2.44)	0.062	1.63 (1.04; 2.56)	0.036
ANC2							
Log CRP, ± SD	239	0.5 ± 0.7	0.9 ± 0.6	2.11 (1.25; 3.58)	0.004	1.78 (1.05; 3.00)	0.023
CRP > 5, n/N (%)	239	74/208 (36)	19/31 (61)	2.49 (1.26; 4.90)	0.007	2.06 (1.04; 4.10)	0.034
CRP > 10, n/N (%)	239	49/208 (24)	12/31 (38)	1.84 (0.95; 3.58)	0.082	1.48 (0.75; 2.91)	0.26

Parameter mg/l	n	Outcome^a^		Difference or relative risk^a^ (95% CI)	P	Adjusted difference or relative Risk^b^ (95% CI)	P_adj_^c^
		AGA	SGA				
Baseline							
Log CRP, ± SD	271	− 0.2 ± 0.7	− 0.1 ± 0.7	1.06 (0.83; 1.35)	0.67	1.10 (0.86; 1.4)	0.51
CRP > 5, n/N (%)	271	15/183 (8)	11/88 (13)	1.35 (0.83; 2.19)	0.27	1.37 (0.86; 2.17)	0.23
CRP > 10 n/N (%)	271	7/183 (4)	5/88 (6)	1.30 (0.65; 2.61)	0.50	1.41 (0.76; 2.63)	0.37
ANC1							
Log CRP, ± SD	273	0.7 ± 0.7	0.4 ± 0.7	0.75 (0.60; 0.94)	0.012	0.77 (0.61; 0.96)	0.018
CRP > 5, n/N (%)	273	103/185 (56)	33/88 (38)	0.60 (0.42; 0.87)	0.005	0.62 (0.44; 0.09)	0.009
CRP > 10, n/N (%)	273	70/185 (38)	23/88 (26)	0.68 (0.46;1.03)	0.054	0.71 (0.47; 1.07)	0.086
ANC2							
Log CRP, ± SD	236	0.5 ± 0.7	0.6 ± 0.7	1.08 (0.83; 1.41)	0.58	1.10 (0.84; 1.44)	0.51
CRP > 5, n/N (%)	236	61/155 (39)	32/81 (40)	1.00 (0.70; 1.44)	0.98	1.02 (0.70; 1.47)	0.92
CRP > 10, n/N (%)	236	40/155 (26)	21/81 (26)	1.00 (0.67; 1.50)	0.98	1.02 (0.68; 1.54)	0.91

Antenatal clinic visits: ANC1 scheduled at 13–16 weeks gestation; ANC2 at 33–36 weeks gestation

*AGA* appropriate for gestational age, *SGA* small for gestational age

^a^N(%) for categorical variables; mean ± SD for log(CRP)

^b^Relative risk for the categorical variables; difference in log_10_ CRP

^c^Adjusted for assessment month

Genital tract infection biomarkers at enrolment, ANC1, or ANC2 showed no significant associations with preterm/term outcomes (Table S3, Additional File 2), or SGA/AGA outcomes (Additional file 2: Table S4). All women (one missing) had a negative syphilis test at ANC1.

### Seasonal patterns and birth outcomes

**Fig. 4:**
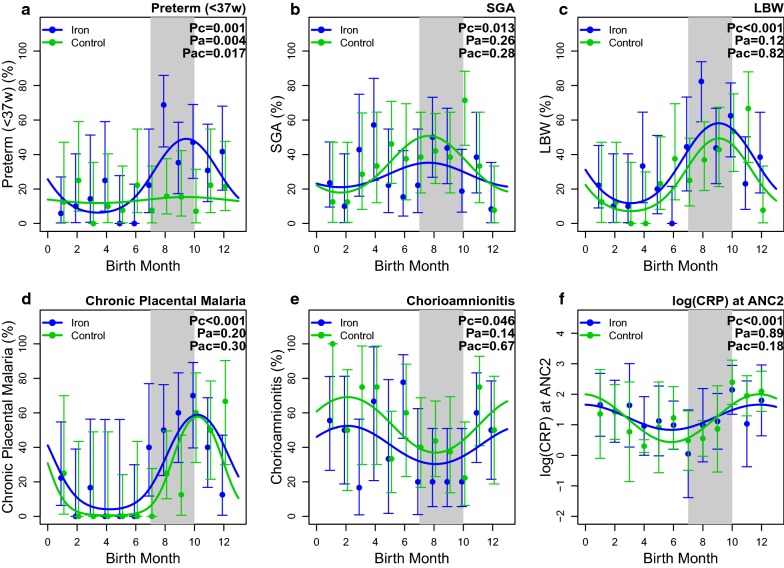
Seasonal patterns and birth outcomes. Seasonal trends by trial arm in **a** PTB; **b** SGA; **c** LBW; **d** chronic placental malaria; **e** chorioamnionitis and **f** CRP at ANC2 assessment. The upper row shows the proportion (with 95% confidence intervals) for PTB, SGA and LBW outcomes by month of birth, with the months May/June to September/October occurring in the rainy season, and the months November through April in the dry season. The shaded region (July–September) represents the main malaria transmission season [13]. The lower panel provides the seasonal patterns by trial arm for chronic placental malaria, chorioamnionitis grades 2 and 3, and log CRP concentration at ANC2. Error bars represent 95% CI. Significance tests for season and arm effects based on a sinusoidal model adjusting for bed net use and baseline MUAC: Pc is a test for a sinusoidal seasonal trend; Pa is a test for difference between arms adjusting for season (birth month); Pac is a test for an interaction between seasonality and arm

Figure 4 shows plots of seasonality giving the two trial arms separately for birth months between January and December. The birth and assessment month distributions were similar between trial arms. PTB incidence showed a striking difference in seasonality, mainly in those born in the malaria transmission period towards the end of the year. Women receiving iron showed not only a higher mean rate (P = 0.001) but also a stronger seasonal effect (interaction test P = 0.017) with peak PTB incidence of approximately 50%, compared to less than 20% in controls.

SGA showed a weaker seasonality with a definite low period at the end of the year, but with no evidence of arm differences (P = 0.28). Low birthweight (LBW) combines PTB and SGA and showed a strong, clear, seasonal effect (P < 0.001), with no significant difference between trial arms (P = 0.82).

The seasonal analysis showed a marked cyclic pattern for chronic placental malaria (P < 0.001) with peak prevalence months late in the year, but with no significant difference by trial arm (P = 0.30) (Fig. 4). For chorioamnionitis (grades 2 and 3) the seasonal pattern (P = 0.046) was less marked and peak prevalence occurred in the early drier months of the year, with no difference by trial arm (P = 0.67). Seasonal variation was also observed for maternal CRP concentration in late pregnancy (P < 0.001), with a peak for those delivering late in the year, and no difference by trial arm.

### Placental pathology in relation to PTB or SGA

Similar numbers of placental biopsies (n = 86 and 92) were available for each trial arm. Placental samples were not available for home deliveries or those *en route* to hospital, for retained placenta, or where maternal/newborn illness at delivery made collection difficult. Chronic placental malaria was associated with a 2.00 (95% CI 1.06:3.78) fold increased risk of PTB unadjusted for season (41.9% vs 23.3%, P = 0.039), and a risk ratio of 1.49 (95% CI 0.75:2.94), after adjustment for season (P_adj_ = 0.26), (Table 4). Acute or chronic placental malaria were not significantly associated with SGA risk. Chorioamnionitis (grades 2 or 3) was not significantly associated with either outcome.

**Table 4:** Placental pathology by preterm/term and AGA/SGA categories

Placental pathology n/N (%)^a^	n	Term	Preterm	Relative risk (95% CI)	P	Adjusted relative risk (95% CI)	P_adj_^b^
Acute malaria	181	10/150 (6.7)	2/31 (6.5)	0.97 (0.26; 3.62)	0.97	0.99 (0.27; 3.66)	0.99
Chronic malaria	181	35/150 (23.3)	13/31 (41.9)	2.00 (1.06; 3.78)	0.039	1.49 (0.75; 2.94)	0.26
Past malaria	181	81/150 (54.0)	15/31 (48.4)	0.83 (0.44; 1.58)	0.57	1.08 (0.56; 2.08)	0.82
Chorioamnionitis grade 3	179	11/148 (7.4)	2/31 (6.5)	0.88 (0.23; 3.32)	0.85	1.00 (0.25; 4.00)	0.99
Chorioamnionitis grades 2 and 3	179	67/148 (45.3)	13/31 (41.9)	0.89 (0.46; 1.72)	0.73	0.96 (0.49; 1.87)	0.90

Placental pathology n/N (%)^a^	n	AGA	SGA	Relative risk (95% CI)	P	Adjusted relative risk (95% CI)	P_adj_^b^
Acute malaria	179	7/115 (6.1)	5/64 (7.8)	1.18 (0.58; 2.39)	0.66	1.03 (0.53; 1.99)	0.94
Chronic malaria	179	30/115 (26.1)	17/64 (26.6)	1.02 (0.65; 1.59)	0.95	0.80 (0.50; 1.27)	0.32
Past malaria	179	63/115 (54.8)	32/64 (50.0)	0.88 (0.60; 1.31)	0.54	1.06 (0.71; 1.57)	0.80
Chorioamnionitis grade 3	177	9/114 (7.9)	4/63 (6.3)	0.86 (0.37; 1.99)	0.70	1.02 (0.45; 2.27)	0.97
Chorioamnionitis grades 2 and 3	177	54/114 (47.4)	26/63 (41.3)	0.85 (0.57; 1.28)	0.43	1.04 (0.70; 1.54)	0.86

*AGA* appropriate for gestational age, *SGA* small for gestational age

^a^Acute: only parasites and minimal hemozoin deposition in the macrophages but not fibrin; Chronic: parasites and Hemozoin deposition; Past: hemozoin usually mixed with fibrin but no parasites. Severity of acute chorioamnionitis and funiculitis (acute histologic chorioamnionitis) was graded histologically as early (grade 1), intermediate (grade 2) and advanced (grade 3) following the Redline-classification [17, 18]

^b^Adjusted for birth month

## Discussion

Weekly iron and folic acid supplementation, given to young women for up to 18 months preceding pregnancy and until the first antenatal visit, significantly shortened gestational age and increased the incidence of spontaneous PTB at less than 37 weeks. PTB incidence followed a marked seasonal pattern, with a peak incidence of around 50% in women receiving iron supplements, compared with less than 20% in women receiving folic acid alone. This seasonal peak corresponded with that for chronic placental malaria infection towards the end of the rainy season. Chorioamnionitis, another important infectious cause of PTB, was marginally significantly increased in the dry season, but not increased in supplemented women.

Supplement adherence was balanced between trial arms in wet and dry seasons with good weekly adherence (69–100%). Iron deficiency prevalence doubled in both trial arms between ANC1 and ANC2. Iron status improves in early pregnancy in these women compared to their non-pregnant counterparts [15], and an increase in iron deficiency prevalence between ANC1 and ANC2 would be expected if later gestational iron requirements were not met. As the inter-quartile age range was 16–18 years continued linear growth may still be occurring, further increasing iron requirements [21]. IPTp uptake for ≥ two doses was slightly lower in the iron arm, which can primarily be attributed to some deliveries occurring in iron-supplemented women prior to receipt of the second IPTp dose.

Mean gestational age, PTB, birthweight and placental malaria were four of the pre-specified secondary outcomes for the pregnant cohort, and as some of these outcomes admitted multiple time points or definitions a rigorous allowance for multiple testing is not possible. However, the significance level was such (P < 0.001 for PTB) that these results are unlikely to be chance findings. Nevertheless, some caution is required, and further studies are needed to confirm these findings. A trial limitation was attrition due to out-migration from the study area, leading to delivery attendance outside the study location. However, attrition was equivalent between trial arms and its level unsurprising given the long period of follow-up, and the adolescent profile of a culture in which young women move with their husbands at the time of marriage.

Genital tract infection prevalence (*Trichomonas vaginalis*, BV), which can increase chorioamnionitis and/or PTB risk [22], was equivalent between trial arms at ANC1 as previously reported [9]. Exploratory analyses showed no significant association of *Trichomonas vaginalis*, BV or chorioamnionitis with PTB. As a young population, women were only recently exposed to regular sexual activity and risk of sexually transmitted infections, and syphilis prevalence was zero at ANC1 [8]. Small stature is considered a risk factor for term SGA and PTB [23], but there were no baseline differences between trial arms in maternal stature, or change in mean height between baseline and ANC1. All women were primigravidae, and over 90% were adolescent, an important risk factor for PTB [24]. As mean age and the proportion of younger women (< 17 years) were almost identical between arms, this is an unlikely source of bias. Hypertension, which occurs more commonly in young nulliparae and is associated with PTB, was infrequent, occurring in only two women (0.7%), which is at the lower prevalence limit for children and adolescents in Western Africa (range 0.2–6.3%) [25].

The gestational age distribution curve was shifted to the left in iron-supplemented women suggesting a population effect had resulted in more preterm deliveries. Population estimates for prevalence of preterm delivery in sub-Saharan Africa in 2014 range from 8.6 to 16.7% [26], and are lower than the mean prevalence of 20.9% reported here. Previous estimates from malaria endemic countries range from 6.4 to 9.4% [27], but were not based on ultrasound measurement, which is required for accurate assessment [28]. Compared with studies in sub-Saharan Africa that used ultrasound, mean gestational age in iron-supplemented women in the present trial was 10.4 days shorter than in Tanzanian women (mainly older multigravidae) receiving daily antenatal iron (274.4 days) [29], and 6.2 days shorter than a population sample of Malawian women (270.2 days) in which young age (< 20 years) and persistent malaria were identified as significant negative risk factors [30]. In Malian primigravidae with malaria infection, mean ultrasound dated gestational age was 268.9 days and PTB prevalence 15.8% [31]. Mean gestational ages in the Malawian and Malian studies were comparable to those of women in the control arm of the present trial (269.4 days). In Burkina Faso SGA did not differ by intervention arm, but the 32.1% incidence was marginally higher than the global estimates for sub-Saharan Africa in 2010 which ranged from 21.7% to 28.8% [27]. Thus, incidence of both PTB and SGA outcomes was high in this trial in both study arms compared to other estimates, and this most likely reflects the young age and nulliparity of the population. This was further exacerbated by iron supplementation, leading to excess PTB in the intervention arm.

There was no reduction in iron deficiency or anaemia at ANC1 in the iron-supplemented cohort, despite good weekly supplement adherence [8, 9]. This was attributed to poor iron absorption resulting from raised hepcidin concentration [32], and tissue-specific gut mucosal damage secondary to chronic malaria [33]. Many women had asymptomatic malaria which, as a result, was untreated [8, 34]. Women receiving iron had received significantly more antibiotic and antifungal prescriptions predominantly for enteric infections [9]. Chronic sequestration of *P. falciparum*-infected erythrocytes within gut endothelium would contribute to gut-barrier dysfunction [35, 36]. A combination of chronic systemic malarial inflammation [31], and chronic enteric infection risk would create a ‘double hit’ mechanism [37]. There was significantly higher plasma CRP concentration, indicating systemic inflammation, in women with PTB, with a risk ratio at ANC2 of 2.06 (95% CI 1.04:4.10) for raised values > 5. By comparison, in a meta-analysis from non-malaria endemic countries, raised plasma CRP was associated in mid-gestation with a risk ratio of 1.53 (95% CI 1.22:1.90) for spontaneous PTB [38]. Systemic [39, 40], and tissue-level inflammation [33], would be expected to increase risk of labour and PTB [41]. However in this study, there were no treatment-associated differences in CRP, which was the only marker of inflammation available. Thus it could not substantiate a CRP-related mechanism, nor estimate direct and inflammation-mediated effects from the data available. Whether this reflects an absence of an inflammation-mediated effect, or a weakness in ascertainment of inflammation, including use of enteric biomarkers, is not clear.

SGA birth was not associated with raised CRP concentrations, and raised values were actually lower at ANC1 for this outcome. Previous studies in non-malaria endemic areas have reported a positive association with fetal growth restriction but only when CRP levels were very high (> 25 mg/l) [42], a weak positive association in late pregnancy [43], or no association [44]. In Papua New Guinea, CRP at enrolment and at delivery in women receiving sulphadoxine-pyrimethamine plus chloroquine (compared to sulphadoxine-pyrimethamine plus azithromycin), was positively associated with PTB, but not SGA [45].

## Conclusions

This trial provides evidence that long-term iron supplementation leads to excess PTB in a malaria endemic area, predominantly associated with the malaria transmission season, with the risk of delivery under 37 weeks of 27.5% compared to 13.9% in non-iron-supplemented primigravidae, and an average gestation 5 days shorter. While underlying mechanisms require further investigation, there is some evidence from the present study which implicates dual infection exposure. Before iron supplementation can be recommended prior to the first pregnancy, malaria control must ensure improved uptake of IPTp-SP with reduction of chronic asymptomatic parasitaemias to avoid increasing risk of subsequent PTB. Early PTB precludes administration of later gestational doses of IPTp reducing protection. Routine iron supplements are not needed in populations, which have low prevalence of iron deficiency, such as in these young women. These findings are particularly relevant to adolescents living in sub-Saharan Africa where perennial malaria transmission occurs and where long-term iron supplementation, as routinely offered to populations such as this, is potentially harmful.

## Additional files

**Additional file 1.** Further trial details. Further details and background to the Trial: Summary of previously published studies on the trial; summary of pregnancy-related adverse events; study area and participants; procedures and randomization; pregnant cohort study assessments; data collection and monitoring; laboratory procedures (iron biomarkers, malaria microscopy, placental sampling), references.

**Additional file 2: Table S1.** Characteristics of women who became pregnant, proceeded to singleton live births and who had assessment at birth. **Table S2.** Results of the ITT analysis comparing CRP between trial arms at ANC1 and ANC2. **Table S3.** Genital tract infection biomarkers at enrolment, ANC1, or ANC2, for preterm/term outcomes. **Table S4.** Genital tract infection biomarkers at enrolment, ANC1, or ANC2, for SGA/AGA outcomes.

## Data Availability

The datasets used and/or analysed during the current study are available from the corresponding author on reasonable request and will be made available following an end user data agreement and sponsor approval.

## Abbreviations

WHO: World Health Organization
ANC1: First scheduled antenatal survey in primigravidae
ANC2: Second scheduled antenatal survey in primigravidae
sTfR: serum transferrin receptor
CRP: C-reactive protein
IPTp: intermittent preventive antimalarial treatment in pregnancy
IPTp-SP: intermittent preventive treatment in pregnancy with sulfadoxine-pyrimethamine
RDT: rapid diagnostic test for malaria
RPR/VDRL: Venereal Disease Research Laboratory Test
qPCR: quantitative polymerase chain reaction
ITT: intention to treat
BV: bacterial vaginosis
SAE: serious adverse event
MUAC: mid-upper-arm-circumference
BMI: body mass index
PTB: preterm birth
LBW: low birthweight
SGA: small for gestational age
AGA: appropriate for gestational age
RR: relative risk
IQR: inter-quartile range
SD: standard deviation

## References

[1] WHO (2016). Guidelines: daily iron supplementation of adult women and adolescent girls.

[2] WHO (2011). Guidelines: intermittent iron and folic acid supplementation in menstruating women.

[3] Low MSY, Speedy J, Styles CE, De-Regil LM, Pasricha SR (2016). Daily iron supplementation for improving anaemia, iron status and health in menstruating women. Cochrane Database Syst Rev..

[4] Clark MA, Goheen MM, Fulford A, Prentice AM, Elnagheeb MA, Patel J (2014). Host iron status and iron supplementation mediate susceptibility to erythrocytic stage *Plasmodium falciparum*. Nat Commun..

[5] Brabin BJ (1983). An analysis of malaria in pregnancy in Africa. Bull World Health Organ.

[6] Brabin L, Brabin BJ, Gies S (2013). Influence of iron status on risk of maternal or neonatal infection and on neonatal mortality with an emphasis on developing countries. Nutr Rev.

[7] Pena-Rosas JP, De-Regil LM, Garcia-Casal MN, Dowswell T (2015). Daily oral iron supplementation during pregnancy. Cochrane Database Syst Rev..

[8] Gies S, Diallo S, Roberts SA, Kazienga A, Powney M, Brabin L (2018). Effects of weekly iron and folic acid supplements on malaria risk in nulliparous women in Burkina Faso: a periconceptional double-blind randomized controlled non-inferiority trial. J Inf Dis.

[9] Brabin L, Roberts SA, Gies S, Nelson A, Diallo S, Stewart CJ (2017). Effects of long-term weekly iron and folic acid supplementation on lower genital tract infection—a double blind, randomised controlled trial in Burkina Faso. BMC Med..

[10] Institut National de la Statistique et de la Démographie (INSD) et ICF International (2012). Enquète Demographique et de Santé et Indicateurs Multiples du Burkina Faso 2010.

[11] Bisseye C, Sanou M, Nagalo BM, Kiba A, Compaoré TR, Tao I (2013). Epidemiology of syphilis in regional blood transfusion centres in Burkina Faso, West Africa. Pan Afr Med J..

[12] Derra K, Rouamba E, Kazienga A, Ouedraogo S, Tahita MC, Sorgho H (2012). Profile: Nanoro health and demographic surveillance system. Int J Epid..

[13] Rouamba T, Nakanabo-Diallo S, Derra K, Rouamba E, Kazienga A, Inoue Y (2019). Socioeconomic and environmental factors associated with malaria hotspots in the Nanoro demographic surveillance area, Burkina Faso. BMC Public Health.

[14] Ballard JL, Khoury JC, Wedig K, Wang L, Eilers-Walsman BL, Lipp R (1991). New Ballard Score, expanded to include extremely premature infants. J Pediatr.

[15] Diallo S, Roberts SA, Gies S, Rouamba T, Swinkels DW, Geurts-Moespot AJ, et al. Malaria early in the first pregnancy: potential impact of iron status. Clin Nutr 2019. pii: S0261-5614(19)30034-2. 10.1016/j.clnu.2019.01.016. 10.1016/j.clnu.2019.01.016 PMC6660428 30737046

[16] Ismail MR, Ordi J, Menendez C, Ventura PJ, Aponte JJ, Kahigwa E (2000). Placental pathology in malaria: a histological, immunohistochemical, and quantitative study. Hum Pathol.

[17] Redline RW (2002). Clinically and biologically relevant patterns of placental inflammation. Pediatr Dev Pathol.

[18] Léger-Rave M-B, Patrier S, Razavi FE, Carles D, Bouvier R, Dauge MC (2008). Les infections foeto-placentaires. Pathologie Fœtale et Placentaire.

[19] Villar J, Cheikh Ismail L, Victora CG, Ohuma EO, Bertino E, Altman DG (2014). International standards for newborn weight, length, and head circumference by gestational age and sex: the Newborn Cross-Sectional Study of the INTERGROWTH-21st Project. Lancet.

[20] R Core Team. R: a language and environment for statistical computing. Vienna, Austria: R Foundation for Statistical Computing, 2016. https://www.R-project.org/. Accessed 2018.

[21] Kalanda BF, Verhoeff FH, Brabin BJ (2006). Chronic malnutrition in pregnant adolescents in rural Malawi: an anthropometric study. Acta Obstet Gynecol Scand.

[22] Kim CJ, Romero R, Chaemsaithong P, Kim JS (2015). Chronic inflammation of the placenta: definition, classification, pathogenesis, and clinical significance. Am J Obstet Gynecol.

[23] Kozuki N, Katz J, Lee AC, Child Health Epidemiology Reference Group (2015). Short maternal stature increases risk of small-for-gestational-age and preterm births in low- and middle-income countries: individual participant data meta-analysis and population attributable fraction. J Nutr..

[24] Mombo-Ngoma G, Mackanga JR, González R, Ouedraogo S, Kakolwa MA, Manego RZ (2016). Young adolescent girls are at high risk for adverse pregnancy outcomes in sub-Saharan Africa: an observational multi country study. BMJ Open.

[25] Noubiap JJ, Essouma M, Bigna JJ, Jingi AM, Aminde LN, Nansseu JR (2017). Prevalence of elevated blood pressure in children and adolescents in Africa: a systematic review and meta-analysis. Lancet Public Health..

[26] Chawanpaiboon S, Vogel JP, Moller AB, Lumbiganon P, Petzold M, Hogan D (2019). Global, regional, and national estimates of levels of preterm birth in 2014: a systematic review and modelling analysis. Lancet Glob Health.

[27] Lee AC, Katz J, Blencowe H, CHERG Working Group (2013). SGA-Preterm Birth Working Group National and regional estimates of term and preterm babies born small for gestational age in 138 low-income and middle-income countries in 2010. Lancet Glob Health.

[28] Rijken MJ, De Livera AM, Lee SJ, Boel ME, Rungwilailaekhiri S, Wiladphaingern J (2014). Quantifying low birth weight, preterm birth and small-for-gestational-age effects of malaria in pregnancy: a population cohort study. PLoS ONE.

[29] Mwangi MN, Roth JM, Smit MR, Trijsburg L, Mwangi AM, Demir AY (2015). Effect of daily antenatal iron supplementation on Plasmodium infection in Kenyan women: a randomized clinical trial. JAMA.

[30] Van den Broek NR, Jean-Baptiste R, Neilson JP (2014). Factors associated with preterm, early preterm and late preterm birth in Malawi. PLoS ONE.

[31] Fried M, Kurtis JD, Swihart B, Pond-Tor S, Barry A, Sidibe Y (2017). Systemic inflammatory response to malaria during pregnancy is associated with pregnancy loss and preterm delivery. Clin Infect Dis.

[32] Spottiswoode N, Duffy PE, Drakesmith H (2014). Iron, anemia and hepcidin in malaria. Front Pharmacol..

[33] Coban C, Lee MSJ, Ishii KJ (2018). Tissue-specific immunopathology during malaria infection. Nat Rev Immunol.

[34] Berry I, Walker P, Tagbor H, Bojang K, Coulibaly SO, Kayentao K (2018). Seasonal dynamics of malaria in pregnancy in West Africa: evidence for carriage of infections acquired before pregnancy until first contact with antenatal care. Am J Trop Med Hyg.

[35] Verma S, Cherayil BJ (2017). Iron and inflammation—the gut reaction. Metallomics..

[36] Odenwald MA, Turner JR (2017). The intestinal epithelial barrier: a therapeutic target?. Nat Rev Gastroenterol Hepatol..

[37] Cardenas I, Mor G, Aldo P, Koga K, Lang SM, Booth CJ (2011). Placental viral infection sensitizes to endotoxin-induced pre-term labor: a double hit hypothesis. Am J Reprod Immunol.

[38] Wei S-Q, Fraser W, Luo Z-C (2010). Inflammatory cytokines and spontaneous preterm birth in asymptomatic women, a systematic review. Obs Gynecol..

[39] Peters GA, Yi L, Skomorovska-Prokvolit Y, Patel B, Amini P, Tan H, Mesiano S (2017). Inflammatory stimuli increase progesterone receptor-A stability and transrepressive activity in myometrial cells. Endocrinology.

[40] Talati AN, Hackney DN, Mesiano S (2017). Pathophysiology of preterm labor with intact membranes. Semin Perinatol.

[41] Sandman CA, Glynn L, Schetter CD, Wadhwa P, Garite T, Chicz-DeMet A (2006). Elevated maternal cortisol early in pregnancy predicts third trimester levels of placental corticotropin releasing hormone (CRH): priming the placental clock. Peptides.

[42] Ernst GD, de Jonge LL, Hofman A, Lindemans J, Russcher H, Steegers EA (2011). C-reactive protein levels in early pregnancy, fetal growth patterns, and the risk for neonatal complications: the Generation R Study. Am J Obstet Gynecol.

[43] Ferguson KK, Kamai EM, Cantonwine DE, Mukherjee B, Meeker JD, McElrath TF (2018). Associations between repeated ultrasound measures of fetal growth and biomarkers of maternal oxidative stress and inflammation in pregnancy. Am J Reprod Immunol.

[44] Erkenekli K, Keskin U, Uysal B, Kurt YG, Sadir S, Çayci T (2015). Levels of neopterin and C-reactive protein in pregnant women with fetal growth restriction. J Obstet Gynaecol.

[45] Unger HW, Hansa AP, Buffet C, Hasang W, Teo A, Randall L (2019). Sulphadoxine-pyrimethamine plus azithromycin may improve birth outcomes through impacts on inflammation and placental angiogenesis independent of malarial infection. Sci Rep..

