# Supplementary material for: Excess risk of preterm birth with periconceptional iron supplementation in a malaria endemic area: analysis of secondary data on birth outcomes in a double blind randomized controlled safety trial in Burkina Faso
Source: Malar J. 2019 May 6;18:161. doi: 10.1186/s12936-019-2797-8 (PMC6501288; doi:10.1186/s12936-019-2797-8)
Supplement: Supplementary file 1 — Additional file 1. Further trial details. Further details and background to the Trial: Summary of previously published studies on the trial; summary of pregnancy-related adverse events; study area and participants; procedures and randomization; pregnant cohort study assessments; data collection and monitoring; laboratory procedures (iron biomarkers, malaria microscopy, placental sampling), references. [file 12936_2019_2797_MOESM1_ESM.docx]

Additional File 1: Further trial details

**List of contents**

1. **Study area and participants**
2. **Field procedures**
3. **Pregnant cohort study assessments**
4. **Data collection and monitoring**
5. **Laboratory procedures**

- **Iron biomarkers**
- **Malaria microscopy**
- **Placental sampling**

1. **Summary of previously published studies on this trial and results**
2. **References**

***a. Study area and participants:*** The Health and Demographic Surveillance System (HDSS) area was established in 2009. By December 2011, the HDSS recorded approximately 61 000 inhabitants, predominantly of Mossi origin. Health care was provided by twelve peripheral health centres and one referral hospital [1].

Individual participant information about the trial was provided by a study investigator who read this to women who were illiterate before signed consent (part 1) was obtained. Unmarried minor girls were accompanied by a legal representative who agreed to their participation. A second signed consent procedure (part 2) was instigated for participants who became pregnant during the trial, who agreed to attend for a study antenatal visit and to continue weekly follow-up visits.

**b*. Field procedures****:* At trial enrolment (ENR) demographic data and a general history of past and present illnesses and obstetrical history including last menstrual period, age at menarche, sexual activity, use of contraceptive methods and current complaints were recorded. A study clinician completed a physical and clinical examination, including duplicate measurements of height (nearest mm, Minimeter, Raven Equipment Ltd, Essex, UK), weight (nearest 100gms, SECA scale), and mid-upper arm circumference (mm, MUAC). Women were not recruited if they had any significant illness at the time of screening that required hospitalisation, including clinical signs of severe anaemia (conjunctival or mucosal pallor, tachycardia, respiratory distress), or a history or presence of major clinical disease likely to influence pregnancy outcome (sickle cell disease, diabetes mellitus, severe renal or heart disease, open tuberculosis, epilepsy, known HIV/AIDS infection). One self-taken vaginal sample for a BV glass slide was obtained using a cotton-tipped swab in a sterile tube (TSC, Lancs, UK), and a second swab for pH measurement (pH indicator sticks ranged from 3.6-6.1). Women with signs and symptoms of vaginal infections were treated.^3^ All participants received a single dose of albendazole (400mg) and praziquantel (1500-2400mg, according to the WHO dose pole) plus an insecticide treated bed net. Following recruitment participants were individually randomised to one of two trial arms and received weekly one of identical red coloured vegetable cellulose (hypromellose) capsules (disintegration time less than 30 minutes, mean 9.5 minutes), containing either ferrous gluconate (60mg) and folic acid (2.8mg), or folic acid alone (2.8mg) (G and G Food Supplies Ltd, West Sussex, UK), which were provided in tamper evident opaque containers of 20 capsules. The dose of supplements was based on recommendations made by WHO. Participants were individually randomised to receive weekly one of identical red colored vegetable cellulose (hypromellose) capsules (disintegration time <30 minutes, mean 9.5 minutes), containing either ferrous gluconate (60 mg elemental iron, 479 mg gluconate) and folic acid 2.8 mg, or folic acid alone. A block allocation sequence was used with randomly determined block lengths. Four containers (4x20 capsules) were assigned the same randomisation code and were allocated to each participant. The FFA kept one container per participant, obtaining a replacement as required. Supplements were not kept by participants. Supplement codes, unknown to investigators and maintained independently by the sponsor, were revealed only after data base lock and completion of data analysis. Women received a card with a unique study number corresponding to the randomization list. Supplements were stored at 20 -25^0^C and at ambient temperature while with FFAs.

**c*. Pregnant cohort study assessments****:* Women who became pregnant within the 18 month follow up period and consented to enter the pregnancy cohort were referred to Nanoro hospital for a scheduled antenatal visit (standardised at about 13-16 weeks gestation according to the last menstrual period). This was termed ANC1 and was performed by one of the study nurses/doctors and gestational age was confirmed by ultrasound examination. For one baby ultrasound measurement was not available and gestation was determined by physical examination at birth using the Ballard method [2]. The weekly supplement was withdrawn when antenatal care commenced. Laboratory procedures and haematinics provided according to national policy as daily iron (60mg elemental iron) and folic acid (400µg) tablets, but weekly follow-up continued. Routine antenatal care included antenatal booklet, screening for pre-eclampsia, clinical anaemia and syphilis, counselling and voluntary HIV testing and tetanus toxoid immunisation. All women regardless of the malaria laboratory result received the routine first dose of the anti-malarial sulfadoxine-pyrimethamine (IPTp-SP) if gestational age was >13 weeks. Women in the first trimester (≤13 weeks gestation), if malaria positive, were treated with oral quinine. Severely anaemic pregnant (Hb <7g/dL) women were referred to Nanoro hospital. At ANC1 women were treated symptomatically for BV and *T. vaginalis* [3]. Women then followed routine antenatal visits in their respective health centres where they received a second dose of IPTp-SP which was recorded in the ANC booklet.

**d*. Data collection and monitoring***

The study was implemented according to the approved protocol and study specific study operating procedures (SOPs). Questionnaire data was entered directly into an electronic Case Report Form (CRF) on MACRO (InferMed, UK), Good Clinical Practice (GCP) compliant software for clinical trials. Weekly follow-up visits were recorded on electronic questionnaires using Personal Digital Assistant (PDA) handheld devices with in-built consistency checks. PDA data was uploaded weekly onto Macro. An external independent trial monitor from the Institute of Tropical Medicine in Antwerp assessed SOP adherence and reported to the Sponsor on GCP compliance and trial conduct on three occasions. An internal monitor not involved in this trial, verified on a continuous basis that the rights and well-being of human subjects were protected and that the trial was conducted in compliance with the approved protocol on a six monthly basis. A Data Safety Monitoring Board met four times during the course of the trial.

**e*. Laboratory procedures***

*Iron biomarkers*

Blood was transported to the research laboratory and within 3 hours, centrifuged, aliquoted and stored at -80°C. Haemoglobin was measured (Sysmex automated analyser) on fresh whole blood. Plasma ferritin and sTfR were calculated using mean values from duplicate ELISA samples (Spectro Ferritin S-22 and TFC 94 TfR, RAMCO Inc). CRP was assessed by ELISA (EU59131IBL, GmbH) and completed sequentially between December 2011 and December 2013. Intra-assay coefficients of variation (CVs) were all < 10%. Ranges for normal controls were: ferritin, 69.1-114.7 µg/l; sTfR, 4.2-5.9 mg/l; CRP, 5-8 mg/l. Definitions of iron deficiency were (1) adjusted ferritin (adjFE) allowing for inflammation, ferritin < 15 μg/L if CRP < 10 mg/L, or ferritin < 70 μg/L if CRP ≥ 10 mg/L; or (2) a ratio of sTfR (mg/L) to log_10_ ferritin (µg/L) > 5.6, which assesses both stored and functional iron and is possibly less affected by inflammation [4].

*Malaria microscopy*

Blood films were stained with Giemsa and read independently by two qualified microscopists and, in the case of discordant results, by a third reader [5].

*Placental sampling*

After the delivery of the placenta, the placenta was placed in a recipient with the fetal side upwards. Using scissors a biopsy of 2,5 cm x 1 cm was excised at mid-distance between the insertion of the umbilical cord and the placenta border and placed into a prefilled specimen container with 10% neutral buffered formalin (CellStor Pot, CellPath Ltd. Newtown SY16 4LE, UK). A 1 cm cross-section from the umbilical cord was cut with scissors at about 5 cm from the insertion and placed into the same recipient. After turning the placenta in order to expose the maternal side upwards, a second biopsy of about the same size was excised at half distance between the center and the border of the placenta and placed into a second container with formalin. A 10 cm x 10 cm piece of the membrane was cut with scissors and placed into the same container.

Formalin fixed specimen were stored for up to 3 months at room temperature in an air-conditioned room (20°C) at the CRUN laboratory. After transport to the department of pathology at the National University Hospital Yalgado Ouedraogo in Ouagadougou, tissue samples were processed by experienced technicians according to standard histopathological procedures.

Samples were embedded in paraffin wax following standard methods. Membranes were rolled to cylinders in order to obtain sufficient material to be cut and embedded. For each set of samples, paraffin sections 3 to 5 µm thick were placed on 2 slides, the fetal side of the placenta together with the cord on one slide, the maternal side of the placenta together with the membrane on another slide. Slides were prepared in duplicate to allow for different staining. De-paraffinised sections were stained with haematoxylin and eosin (H&E) and Giemsa stain. Histological examination of all samples was done by a specialised senior pathologist with light microscopy and under polarised light. Histological classification of placental malaria was based on the different significance of haemozoin and parasitised RBC in the inter-villous space of the maternal side of the placenta according as described by Ismail et al [6].

- Acute infection (only parasites and minimal haemozoin deposition in the macrophages but not fibrin);

- Chronic infection (parasites and haemozoin deposition);

- Past infection (haemozoin usually mixed with fibrin but no parasites);

- No infection.

Severity of acute chorioamniotitis and funiculitis were graded histologically as early, intermediate and advanced following the Redline-classification [7, 8].

***f. Summary of previously published studies on this trial and results:***

1. Trial design. The profile of trial activities evaluating weekly iron supplementation in this trial are outlined in table 7 of this paper [9].
2. Malaria and anaemia outcomes at ANC1*.* Weekly iron supplementation did not increase malaria risk, improve iron status, or reduce anemia. There was no difference at ANC1 in parasitemia prevalence (iron, 53.4% [95% confidence interval {CI}, 45.7%–61.0%]; control, 55.3% [95% CI, 47.3%–62.9%]; prevalence ratio, 0.97 [95% CI, 0.79–1.18]; P = .82), anemia (adjusted effect, 0.96 [95% CI, 0.83–1.10]; P = .52), iron deficiency (adjusted risk ratio [aRR], 0.84 [95% CI, .46–1.54]; P = .58), or plasma iron biomarkers [5].
3. Genital infection outcomes at ANC1. Bacterial vaginosis (BV) prevalence was 7.0%, and *T. vaginalis* prevalence 12.9%. BV and *T. vaginalis* prevalence did not differ between trial arms at ANC1. Iron-supplemented women when non-pregnant received more antibiotic treatments for non-genital infections (P = 0.014; mainly gastrointestinal infections (P = 0.005), and anti-fungal treatments for genital infections (P = 0.014) [3].
4. Iron biomarkers at ANC1. Most biomarkers indicated reduced iron deficiency in early pregnancy, with the exception of haemoglobin. Body iron increased by 0.6 to 1.2 mg/kg in early gestation, did not differ by malaria status in nulliparae, but was higher in primigravidae with malaria (6.5 mg/kg versus 5.0 mg/kg; relative risk 1.53, 95% CI 0.67-2.38, P<0.001) [4].
5. ***References***

[1] Derra K, Rouamba E, Kazienga A, Ouedraogo S, Tahita MC, Sorgho H, et al. Profile: Nanoro health and demographic surveillance system. Int J Epidemiol 2012; 41:1293–301.

[2] Ballard JL, Khoury JC, Wedig K, Wang L, Eilers-Walsman BL, Lipp R. New Ballard Score, expanded to include extremely premature infants. J Pediatrics 1991; 119:417-423.

[3] Brabin L, Roberts SA, Gies S, Nelson A, Diallo S, Stewart CJ, et al. Effects of long-term weekly iron and folic acid supplementation on lower genital tract infection – a double blind, randomised controlled trial in Burkina Faso. BMC Medicine 2017; 15:206:doi10.1186/s12916-017-0967.

[4] Diallo S, Roberts SA, Gies S, Rouamba T, Swinkels DW, Geurts-Moespot AJ, et al. Malaria early in the first pregnancy: potential impact of iron status. Clinical Nutrition 2019; https://doi.org/ 10.1016/j.clnu.2019.01.016.

[5] Gies S, Diallo S, Roberts SA, Kazienga A, Powney M, Brabin L, et al. Effects of weekly iron and folic acid supplements on malaria risk in nulliparous women in Burkina Faso: a periconceptional double-blind randomized controlled non-inferiority trial. J Inf Dis 2018; doi: 10.1093/infdis/jiy257.

[6] Ismail MR, Ordi J, Menendez C, Ventura PJ, Aponte JJ, Kahigwa E, et al. Placental pathology in malaria: a histological, immunohistochemical, and quantitative study. Hum Pathol 2000; 31:85-93.

[7] Redline, R. Pediatr. Dev. Pathol 2002; 5:326.

[8] Léger-Ravet M-B, Patrier S, Les infections foeto-placentaires. In Férechté E. Razavi, Dominique Carles, Pathologie Foetale et Placentaire Pratique, Chapter 15-5, Sauramps Medical, 2008.

[9] Brabin BJ, Gies S, Owens S, Claeys Y, D’Alessandro U, Tinto H, Brabin L. Perspectives on the design and methodology of periconceptional nutrient supplementation trials. Trials 2016; 17:58, DOI: 10.1186/s13063-015-1124-0.
