# Supplementary material for: Excess risk of preterm birth with periconceptional iron supplementation in a malaria endemic area: analysis of secondary data on birth outcomes in a double blind randomized controlled safety trial in Burkina Faso
Source: Malar J. 2019 May 6;18:161. doi: 10.1186/s12936-019-2797-8 (PMC6501288; doi:10.1186/s12936-019-2797-8)
Supplement: Supplementary file 2 — Additional file 2: Table S1. Characteristics of women who became pregnant, proceeded to singleton live births and who had assessment at birth. Table S2. Results of the ITT analysis comparing CRP between trial arms at ANC1 and ANC2. Table S3. Genital tract infection biomarkers at enrolment, ANC1, or ANC2, for preterm/term outcomes. Table S4. Genital tract infection biomarkers at enrolment, ANC1, or ANC2, for SGA/AGA outcomes. [file 12936_2019_2797_MOESM2_ESM.docx]

# Additional File 2 Additional Data

**Table S1** shows participants lost from pregnancy cohort were comparable to those who provided birth data. Reasons for loss to follow-up were primarily out-migration for work or marriage, or with husband’s migration, and miscarriage before ANC1 (see Figure 1), [1].

[1] Campaoré A, Gies S, Brabin B, Tinto H, Brabin L. Community approval required for periconceptional adolescent adherence to weekly iron and/or folic acid supplementation: a qualitative study in rural Burkina Faso. Reproductive Health. 2018, 15:48 DOI.org/10.1186/s12978-018-0490-y.

**Table S2** shows the results of the ITT analysis comparing CRP between trial arms at ANC1 and ANC2

**Tables S3 and S4** show the results of exploratory analyses of relationships between vaginal infection markers assessed at baseline and antenatal visits (ANC1 and ANC2), and preterm birth or small for gestational age outcomes.

## **Table S1** **Characteristics of women who became pregnant, proceeded to singleton live births (excluding miscarriages, stillbirths and lost to follow-up) and who had assessment at birth (excluding those migrating out of area).** See also Figure 1 main paper.

| **Characteristic** | **Pregnant**  **N=478** | **Singleton Live Births**  **All**  **N=433** | **Singleton Live Births Assessed**  **N=307** |
| --- | --- | --- | --- |
| **Allocation** | | | |
| Iron, n/N (%) | 258/478 (54) | 231/433 (53.3) | 163/307 (53.1) |
| Control, n/N (%) | 220/478 (46) | 202/433 (46.7) | 144/307 (46.9) |
| **Socio-demographic, n/N (%)** | | | |
| Age, years, mean (SD) | 17.0 [16.0-18.0] | 17.0 [16.0-18.0] | 17.0 [16.0-18.0] |
| Age <20 years | 438/478 (91.6) | 399/433 (92.1) | 279/307 (90.9) |
| Age <17 years | 194/478 (40.6) | 178/433 (41.1) | 127/307 (41.4) |
| Ethnic Group Mossi | 464/478 (97.1) | 422/433 (97.5) | 300/307 (97.7) |
| Religion: Catholic | 204/477 (42.8) | 181/432 (41.9) | 130/306 (42.5) |
| Protestant | 37/477 (7.8) | 34/432 (7.9) | 23/306 (7.5) |
| Muslim | 127/477 (26.6) | 121/432 (28) | 85/306 (27.8) |
| Traditional | 109/477 (22.9) | 96/432 (22.2) | 68/306 (22.2) |
| Missing | 1/478 (0.2) | 1/433 (0.2) | 1/307 (0.3) |
| No schooling | 318/477 (66.7) | 290/432 (67.1) | 200/306 (65.4) |
| Primary education | 92/477 (19.3) | 82/432 (19) | 56/306 (18.3) |
| Lower Secondary | 62/477 (13) | 55/432 (12.7) | 45/306 (14.7) |
| Higher Secondary | 5/477 (1) | 5/432 (1.2) | 5/306 (1.6) |
| Literate | 130/473 (27.5) | 115/428 (26.9) | 91/304 (29.9) |
| Occupation: ^a^ Student | 111/478 (23.2) | 99/433 (22.9) | 80/307 (26.1) |
| Trading | 20/478 (4.2) | 17/433 (3.9) | 14/307 (4.6) |
| Domestic | 274/478 (57.3) | 251/433 (58) | 175/307 (57) |
| Farming | 231/478 (48.3) | 214/433 (49.4) | 133/307 (43.3) |
| **Other** | 6/478 (1.3) | 5/433 (1.2) | 5/307 (1.6) |
| **Clinical, n/N (%)** |  |  |  |
| Menarcheal | 444/478 (92.9) | 404/433 (93.3) | 288/307 (93.8) |
| Sexually Active | 180/478 (37.7) | 159/433 (36.7) | 115/307 (37.5) |
| Height, cm [IQR] | 159.6 [155.6-163.4] | 159.8 [155.6-163.5] | 159.7 [155.2-163.4] |
| Weight, kg [IQR] | 51.4 [47.5-55.7] | 51.3 [47.5-55.5] | 51.2 [47.4-55.0] |
| BMI, kg/m^2^ [IQR] | 20.2 [18.9-21.4] | 20.1 [18.9-21.3] | 20.0 [18.9-21.0] |
| MUAC, cm [IQR] | 85/478 (17.8) | 80/433 (18.5) | 57/307 (18.6) |
| Height, cm [IQR] | 24.1 [22.9-25.4] | 24.1 [22.9-25.4] | 24.1 [22.9-25.3] |
| **Serum Iron Biomarkers n/N (%)** | | | |
| GM CRP, mg/l [IQR] | 0.71 [0.24-1.65] | 0.71 [0.23-1.65] | 0.65 [0.24-1.68] |
| Missing | 9/478 (1.9) | 8/433 (1.8) | 7/307 (2.3) |
| CRP >5 mg/l | 40/469 (8.5) | 37/425 (8.7) | 27/300 (9) |
| CRP >10mg/l | 19/469 (4.1) | 17/425 (4.0) | 12/300 (4.0) |
| GM Ferritin, µg/l [IQR] | 46.00 [26.00-79.00] | 46.00 [26.25-78.00] | 50.00 [26.00-84.00] |
| Missing | 7/478 (1.5) | 7/433 (1.6) | 6/307 (2.0) |
| GM sTfR, mg/l [IQR] | 6.31 [5.12-7.74] | 6.38 [5.16-7.78] | 6.22 [5.12-7.76] |
| Missing | 7/478 (1.5) | 7/433 (1.6) | 6/307 (2.0) |
| GM sTfR/log_10_ ferritin ratio | 3.82 [2.90-5.35] | 3.87 [2.91-5.42] | 3.80 [2.88-5.58] |
| Missing | 8/478 (1.7) | 8/433 (1.8) | 7/307 (2.3) |
| Iron deficiency (adjusted ferritin) ^b^ | 51/467 (10.9) | 48/423 (11.3) | 37/298 (12.4) |
| Missing | 11/478 (2.3) | 10/433 (2.3) | 9/307 (2.9) |
| Iron deficiency (sTfR/log ferritin ratio >5.6) | 107/470 (22.8) | 102/425 (24) | 75/300 (25) |
| Missing | 8/478 (1.7) | 8/433 (1.8) | 7/307 (2.3) |
| **Infant n/N (%)** | | | |
| Sex: Male | 152/302 (50.3) | 152/302 (50.3) | 152/302 (50.3) |
| **Antenatal Care n/N (%)** | | | |
| First visit (ANC1) | 315/478 (65.9) | 308/433 (71.1) | 286/307 (93.2) |
| Second visit (ANC2) | 247/478 (51.7) | 245/433 (56.6) | 243/307 (79.2) |
| Median total ANC visits [IQR] ^c^ | 4.00 [3.00-5.00] | 4.00 [3.00-5.00] | 4.00 [3.00-5.00] |
| Median IPTp doses, IQR ^d^ | 2.00 [1.00-2.00] | 2.00 [1.00-2.00] | 2.00 [1.00-2.00] |
| ≥ One IPTp dose | 293/307 (95.4) | 293/307 (95.4) | 293/307 (95.4) |
| ≥ Two IPTp dose | 223/307 (72.6) | 223/307 (72.6) | 223/307 (72.6) |

^a^  More than one response allowed

^b^ Ferritin < 15 μg/L if CRP < 10 mg/L, or ferritin < 70 μg/L if CRP ≥ 10 mg/L.

^c^ ANC1, ANC2 and non-study ANC visits.

^d^ IPTp: Intermittent preventive treatment with sulfadoxine-pyrimethamine.

**Table S2: ITT analysis of CRP at the two ANC assessment points by trial arm**

|  |  |  |  | **Unadjusted** | | **Adjusted** | |
| --- | --- | --- | --- | --- | --- | --- | --- |
|  | **n** | **Iron** | **Control** | **Difference** | **P** | **Difference** | **P_adj_** |
| **Adjusted for baseline MUAC, bed net, assessment month** | | | | | | | |
| log_10_(CRP) at ANC1 | 305 | 0.56±0.75  [73 missing] | 0.61±0.70  [55 missing] | -0.05 (-0.21;0.12) | 0.56 | -0.05 (-0.21;0.1) | 0.51 |
| log_10_(CRP) at ANC2 | 241 | 0.56±0.68 [108 missing] | 0.51±0.68  [84 missing] | 0.06 (-0.11;0.23) | 0.50 | 0.01 (-0.16;0.18) | 0.91 |
| **Adjust for month only** ^a^ | | | | | | | |
| log_10_(CRP) at ANC1 | 305 | 0.56±0.75  [73 missing] | 0.61±0.70  [55 missing] | -0.05 (-0.21;0.12) | 0.56 | -0.05 (-0.2;0.11) | 0.55 |
| log_10_(CRP) at ANC2 | 241 | 0.56±0.68 [108 missing] | 0.51±0.68  [84 missing] | 0.06 (-0.11;0.23) | 0.50 | 0.01 (-0.16;0.18) | 0.89 |

^a^ Sensitivity analysis adjusting for month only to mirror that shown in table 3

**Table S3** **Genital tract infection biomarkers at enrolment and antenatal visits ANC1 and ANC2, for preterm/term outcomes**

| **Parameter** | **n** | **Outcome**  **(%)** | | **Relative Risk**  **(95%CI)** | **P** | **Adjusted Relative Risk ^a^**  **(95% CI)** | **P_adj_ ^a^** |
| --- | --- | --- | --- | --- | --- | --- | --- |
| **Baseline** |  | **Term** | **Preterm** |  |  |  |  |
| Vaginal discharge | 286 | 4/226 (1.8) | 1/60 (1.7) | 0.95 (0.16;5.62) | 0.956 | 1.05 (0.19-5.86) | 0.961 |
| Vaginal pH ≥ 4.5 | 238 | 96/192 (50) | 16/46 (34.8) | 0.6 (0.34;1.04) | 0.061 | 0.59 (0.34-1.04) | 0.058 |
| Nugent 0-3 ^b^ | 236 | 144/190 (75.8) | 37/46 (80.4) | 1.25 (0.64;2.43) | 0.497 | 1.25 (0.64-2.43) | 0.497 |
| Nugent 4-6 | 236 | 18/190 (9.5) | 4/46 (8.7) | 0.93 (0.36;2.35) | 0.870 | 0.93 (0.36-2.35) | 0.870 |
| Nugent 7-10 | 236 | 28/190 (14.7) | 5/46 (10.9) | 0.75 (0.32;1.77) | 0.486 | 0.75 (0.32-1.77) | 0.486 |
| **ANC1** |  |  |  |  |  |  |  |
| Vaginal discharge | 285 | 17/226 (7.5) | 3/59 (5.1) | 0.71 (0.24;2.08) | 0.499 | 0.73 (0.25-2.11) | 0.536 |
| Vaginal pH ≥ 4.5 | 281 | 129/224 (57.6) | 34/57 (59.6) | 1.07 (0.67;1.72) | 0.778 | 1.07 (0.66-1.71) | 0.788 |
| Nugent 0-3 ^b^ | 270 | 172/216 (79.6) | 44/54 (81.5) | 1.1 (0.59;2.05) | 0.759 | 0.99 (0.53-1.83) | 0.969 |
| Nugent 4-6 | 270 | 28/216 (13) | 5/54 (9.3) | 0.73 (0.31;1.71) | 0.444 | 0.81 (0.35-1.86) | 0.606 |
| Nugent 7-10 | 270 | 16/216 (7.4) | 5/54 (9.3) | 1.21 (0.54;2.72) | 0.656 | 1.32 (0.59-2.97) | 0.515 |
| *T.vaginalis* | 261 | 23/205 (11.2) | 2/56 (3.6) | 0.35 (0.09;1.36) | 0.058 | 0.35 (0.09-1.35) | 0.053 |
| **ANC2** |  |  |  |  |  |  |  |
| Vaginal discharge | 243 | 16/212 (7.5) | 3/31 (9.7) | 1.26 (0.42;3.8) | 0.688 | 1.62 (0.61-4.31) | 0.416 |
| Vaginal pH ≥ 4.5 | 243 | 132/212 (62.3) | 19/31 (61.3) | 0.96 (0.49;1.9) | 0.917 | 1.06 (0.55-2.08) | 0.852 |
| Nugent 0-3 ^b^ | 228 | 171/199 (85.9) | 25/29 (86.2) | 1.02 (0.38;2.75) | 0.968 | 0.93 (0.35-2.43) | 0.880 |
| Nugent 4-6 | 228 | 15/199 (7.5) | 2/29 (6.9) | 0.92 (0.24;3.57) | 0.901 | 1.19 (0.33-4.26) | 0.810 |
| Nugent 7-10 | 228 | 13/199 (6.5) | 2/29 (6.9) | 1.05 (0.27;4.04) | 0.942 | 0.99 (0.26-3.75) | 0.984 |
| *T.vaginalis* | 238 | 22/209 (10.5) | 2/29 (6.9) | 0.66 (0.17;2.63) | 0.524 | 0.49 (0.13-1.94) | 0.246 |

Antenatal clinic visits: ANC1 scheduled at 13-16 weeks gestation; ANC2 at 33-36 weeks gestation

^a^ Adjusted for assessment month.

^b^ Nugent score from gram stains for abnormal flora: 0 -3 is normal; 4 -6 is intermediate; 7 -10 is bacterial vaginosis.

## **Table S4** **Genital tract infection biomarkers at enrolment and antenatal visits ANC1 and ANC2, for SGA/ AGA outcomes**

| **Parameter** | **n** | **Outcome**  **(%)** | | **Relative Risk**  **(95%CI)** | **P** | **Adjusted Relative Risk ^a^**  **(95% CI)** | **P_adj_ ^a^** |
| --- | --- | --- | --- | --- | --- | --- | --- |
| **Baseline** |  | **AGA** | **SGA** |  |  |  |  |
| Vaginal discharge | 277 | 2/171 (1.2) | 3/106 (2.8) | 1.58 (0.76;3.3) | 0.322 | 1.55 (0.76-3.14) | 0.337 |
| Vaginal pH ≥ 4.5 | 234 | 69/147 (46.9) | 41/87 (47.1) | 1 (0.72;1.41) | 0.978 | 1.03 (0.73-1.44) | 0.874 |
| Nugent 0-3 ^b^ | 232 | 114/146 (78.1) | 65/86 (75.6) | 0.92 (0.62;1.35) | 0.662 | 0.91 (0.62-1.33) | 0.624 |
| Nugent 4-6 | 232 | 15/146 (10.3) | 7/86 (8.1) | 0.85 (0.45;1.6) | 0.588 | 0.85 (0.45-1.61) | 0.600 |
| Nugent 7-10 | 232 | 17/146 (11.6) | 14/86 (16.3) | 1.26 (0.82;1.94) | 0.321 | 1.28 (0.83-1.96) | 0.295 |
| **ANC1** |  |  |  |  |  |  |  |
| Vaginal discharge | 276 | 13/171 (7.6) | 6/105 (5.7) | 0.82 (0.41;1.62) | 0.543 | 0.81 (0.41-1.62) | 0.527 |
| Vaginal pH ≥ 4.5 | 272 | 93/168 (55.4) | 65/104 (62.5) | 1.2 (0.88;1.65) | 0.245 | 1.2 (0.88-1.65) | 0.242 |
| Nugent 0-3 ^b^ | 261 | 132/161 (82) | 79/100 (79) | 0.89 (0.62;1.29) | 0.553 | 0.88 (0.61-1.29) | 0.531 |
| Nugent 4-6 | 261 | 17/161 (10.6) | 13/100 (13) | 1.15 (0.74;1.79) | 0.550 | 1.16 (0.74-1.81) | 0.539 |
| Nugent 7-10 | 261 | 12/161 (7.5) | 8/100 (8) | 1.05 (0.6;1.84) | 0.872 | 1.05 (0.6-1.86) | 0.858 |
| *T.vaginalis* | 252 | 15/152 (9.9) | 9/100 (9) | 0.94 (0.55;1.62) | 0.818 | 0.95 (0.55-1.63) | 0.847 |
| **ANC2** |  |  |  |  |  |  |  |
| Vaginal discharge | 240 | 15/144 (10.4) | 4/96 (4.2) | 0.51 (0.21;1.23) | 0.068 | 0.51 (0.21-1.24) | 0.069 |
| Vaginal pH ≥ 4.5 | 240 | 95/144 (66) | 55/96 (57.3) | 0.8 (0.59;1.1) | 0.175 | 0.81 (0.59-1.11) | 0.201 |
| Nugent 0-3 ^b^ | 225 | 113/136 (83.1) | 81/89 (91) | 1.62 (0.87;3.02) | 0.084 | 1.62 (0.87-3.02) | 0.084 |
| Nugent 4-6 | 225 | 10/136 (7.4) | 6/89 (6.7) | 0.94 (0.49;1.82) | 0.861 | 0.95 (0.49-1.84) | 0.882 |
| Nugent 7-10 | 225 | 13/136 (9.6) | 2/89 (2.2) | 0.32 (0.09;1.19) | 0.021 | 0.32 (0.09-1.18) | 0.020 ^c^ |
| *T.vaginalis* | 235 | 15/139 (10.8) | 8/96 (8.3) | 0.84 (0.47;1.5) | 0.530 | 0.82 (0.46-1.48) | 0.488 |

AGA: appropriate for gestational age; SGA: small for gestational age

Antenatal clinic visits: ANC1 scheduled at 13-16 weeks gestation; ANC2 at 33-36 weeks gestation

^a^ Adjusted for assessment month

^b^ Nugent score from gram stains for abnormal flora: 0 -3 is normal; 4 -6 is intermediate; 7 -10 is bacterial vaginosis.

^c^ Given the large number of infection markers tested, and the fact that these were not pre-specified hypothesis tests, the one isolated P=0.02 value should not be considered statistically significant.
